# Nedd8-Activating Enzyme Is a Druggable Host Dependency Factor of Human and Mouse Cytomegalovirus

**DOI:** 10.3390/v13081610

**Published:** 2021-08-14

**Authors:** Yulia Alejandra Flores-Martínez, Vu Thuy Khanh Le-Trilling, Mirko Trilling

**Affiliations:** Institute for Virology, University Hospital Essen, University of Duisburg-Essen, 45147 Essen, Germany; yulia.flores-martinez@stud.uni-due.de (Y.A.F.-M.); khanh.le@uk-essen.de (V.T.K.L.-T.)

**Keywords:** human cytomegalovirus (HCMV), *Murid herpesvirus 1* (MuHV-1), mouse cytomegalovirus (MCMV), nedd8, nedd8-activating enzyme (NAE), cullin RING ubiquitin ligase (CRL), ubiquitination, host dependency factor, TAS4464, MLN4924

## Abstract

Human cytomegalovirus causes diseases in individuals with insufficient immunity. Cytomegaloviruses exploit the ubiquitin proteasome pathway to manipulate the proteome of infected cells. The proteasome degrades ubiquitinated proteins. The family of cullin RING ubiquitin ligases (CRL) regulates the stability of numerous important proteins. If the cullin within the CRL is modified with Nedd8 (“neddylated”), the CRL is enzymatically active, while CRLs lacking Nedd8 modifications are inactive. The Nedd8-activating enzyme (NAE) is indispensable for neddylation. By binding to NAE and inhibiting neddylation, the drug MLN4924 (pevonedistat) causes CRL inactivation and stabilization of CRL target proteins. We showed that MLN4924 elicits potent antiviral activity against cytomegaloviruses, suggesting that NAE might be a druggable host dependency factor (HDF). However, MLN4924 is a nucleoside analog related to AMP, and the antiviral activity of MLN4924 may have been influenced by off-target effects in addition to NAE inhibition. To test if NAE is indeed an HDF, we assessed the novel NAE inhibitor TAS4464 and observed potent antiviral activity against mouse and human cytomegalovirus. Additionally, we raised an MLN4924-resistant cell clone and showed that MLN4924 as well as TAS4464 lose their antiviral activity in these cells. Our results indicate that NAE, the neddylation process, and CRLs are druggable HDFs of cytomegaloviruses.

## 1. Introduction

Human cytomegalovirus (HCMV; Human betaherpesvirus 5 (HHV-5); Taxonomy ID (TaxID) 10359) is the prototypical member of the *Betaherpesvirinae*. Following a primary infection, HCMV inevitably establishes lifelong latency from which it can reactivate once the host experiences episodes of stress and/or compromised immunity. The HCMV sero-prevalence rates depend on age, sex, and the specific socioeconomic environment of a given collective but are usually high, ranging, e.g., from 56.7% in Germany [1] to almost 100% in certain regions in developing countries. Despite the fact that fatal HCMV infections in apparently healthy individuals sporadically occur [2], most primary infections and reactivation events are self-limiting and progress subclinically—at least in healthy adult individuals. However, HCMV infections often become life-threatening in persons with immature, compromised, or senescent immunity who frequently suffer from symptomatic diseases caused by HCMV replication. Particularly relevant are congenital infections in which HCMV is transmitted from the mother to the developing baby before, during, or after birth. Due to the immature status of the developing immune system, HCMV can replicate in these infants, leading to acute diseases and long-term sequelae such as microcephaly, mental retardation, and sensorineural hearing loss [3]. Accordingly, HCMV is one of the most common perinatal infections in developed countries [4] and a major cause of deafness. Despite the high prevalence of childhood diseases caused by HCMV [5], there is insufficient awareness among women of childbearing age regarding the risks of congenital HCMV infections [6].

Due to the immunosuppression caused either by HIV super-infections or required pharmacologic drug regimens, AIDS patients and transplant recipients frequently experience severe HCMV diseases such as retinitis or pneumonia. Accordingly, the overall survival is significantly diminished in hematopoietic transplant recipients if the donor and/or the recipient are infected with HCMV [7]. In contrast with developed countries, in which highly active antiviral therapy (HAART) against HIV is generally available, a considerable burden of HIV-related cytomegalovirus retinitis and blindness still occurs in resource-limited settings, where people either do not have sufficient access to HAART or do not seek early medical support due to the fear of stigmatization [8,9,10]. Taken together, HCMV infections are highly relevant, especially in situations in which the host immune system fails to prevent HCMV-associated diseases.

Despite extraordinary efforts by academia and companies, neither a prophylactic nor a therapeutic vaccine protecting against HCMV infections and/or HCMV-induced diseases has been approved. Effective direct antiviral drugs such as (val-)ganciclovir, foscarnet, (brin-)cidofovir, and letermovir are available and are extremely important in reducing disease burdens caused by HCMV, e.g., in transplant settings. However, the application of anti-HCMV drugs is limited by toxicity [11,12] and potential teratogenic effects [13]. Additionally, long-term drug treatment selects for viral resistance mutations, e.g., in transplant recipients and people living with HIV. Thus, there is a continuous need for novel drug targets and additional compounds eliciting antiviral activity against herpesviruses such as HCMV. As others and we discussed elsewhere (e.g., [14]), there are two fundamentally different strategies for antiviral drugs: (I) direct acting antivirals (DAA) that target viral enzymes and (II) indirect acting antivirals (IAA) that target host dependency factors (HDFs) encoded by the host genome that are essential for virus replication. While the DAA approach is less prone to cytotoxic effects given that viral proteins are targeted, viral resistance usually arises rather quickly (see e.g., [15,16]). Conversely, for IAA strategies, toxicity is a more important concern because host proteins are the targets, but viral resistance is rather unlikely.

Viruses have a split relationship with the host proteome. Certain proteins such as the constituents of ribosomes or the respiratory chain act as HDFs and are indispensable for viral replication, whereas other proteins denoted host restriction factors (HRF)—which contribute to intrinsic, innate, or adaptive immunity—need to be eliminated by the virus to enable efficient virus propagation. In accordance with the importance of the ubiquitin proteasome system (UPS) in terms of regulating the stability and the turnover of most cellular proteins [17,18], it is not very surprising that numerous viruses exploit the UPS for their benefit [19]. In this respect, cytomegaloviruses are no exception [14,20]. Accordingly, the pharmacologic inhibition of ubiquitin (Ub) conjugation (e.g., by PYR-41, an inhibitor of the Ub-activating E1 enzyme) or the proteolytic activity of the proteasome (e.g., by bortezomib or MG-132) reduce HCMV replication [21,22,23,24]. Lysine (K) 48 (K48) poly-Ub conjugation usually targets proteins for proteasomal degradation. Ubiquitination is a complicated process in which Ub is first activated by the Ub-activating E1 enzyme, then transferred to an Ub-conjugating E2 enzyme, and finally, usually catalyzed by an E3 Ub ligase, transferred to a target protein. Thus, E3 enzymes are important since they select the target proteins of the UPS by bridging them to the Ub conjugation machinery [25,26]. A large, evolutionary conserved, and very important family of E3 Ub ligases (UbL) is the cullin RING UbL family (CRL) [27]. CRLs adjust the abundance of various target proteins and modulate crucial steps in cell biology, such as cell proliferation, DNA damage responses, and stress responses. CRLs possess a special regulation mechanism that is evolutionary related to ubiquitination. The cullin backbone of CRLs can be modified by the small Ub-like molecule Nedd8 in a process termed “neddylation” [28,29]. CRLs harboring neddylated cullins are enzymatically active in terms of ubiquitination, whereas CRLs lacking the Nedd8 moiety are sequestered by proteins such as Cand1 and rendered inactive. Thus, Nedd8 conjugation can be regarded as a reversible molecular switch for CRL activities. Given that CRLs regulate fundamental processes such as cell proliferation, they recently gained a lot of interest as drug targets for oncogenic diseases. MLN4924 was described as a first-in-class drug inhibiting CRL-mediated ubiquitination by binding and inhibiting NAE, the first and rate-limiting enzyme of the neddylation pathway [30]. NAE is a complex composed of the regulatory subunit NAE1 and the catalytic subunit UBA3 (also referred to as NAEβ). MLN4924 has been widely studied in vitro and in vivo and is currently under investigation in several clinical phase 1, 2, and 3 studies (e.g., NCT03268954 and NCT04090736).

Others and we provided evidence that CMVs exploit CRLs [14,31,32,33,34,35,36]. Accordingly, we showed that the NAE inhibitor MLN4924, also known as pevonedistat, elicits broad and potent antiviral activity against mouse and human cytomegalovirus [23]. However, MLN4924 (PubChem CID: 16720766) is structurally related to adenosine 5′-monophosphate (AMP) [30]. Thus, we wondered if MLN4924 elicits its antiviral activity by the inhibition of the canonical NAE function and corresponding downstream effects on CRLs and CRL targets, or if it may rather act by other means in herpesvirus-infected cells. Given its similarity to AMP, MLN4924 may, for example, act as a nucleoside analog affecting viral genome amplification or inhibit virus-encoded proteins such as nucleotide-binding proteins. Here, we followed two different strategies to answer these questions: We used the recently described novel NAE inhibitory drug called TAS4464 [37] to test if the block of NAE/CRL activity by another compound would also elicit antiviral activity against cytomegaloviruses. We reasoned that if NAE constitutes a true HDF and the NAE inhibition determines the molecular reason for the antiviral activity of MLN4924, TAS4464 should elicit comparable antiviral activities against MCMV and HCMV. Additionally, we generated an HCMV-permissive cell clone based on the BJ-5ta cell line that was forced to acquired MLN4924 resistance during long-term cultivation in the presence of increasing MLN4924 concentrations. We reasoned that MLN4924 should show significantly impaired if any antiviral activity in MLN4924 resistant cells if NAE is the main target of MLN4924 in terms of antiviral effects against cytomegaloviruses. However, if MLN4924 acts by other means such as directly inhibiting viral enzymes or viral genome amplification, it should retain its antiviral activity against cytomegaloviruses in MLN4924 resistant cells.

## 2. Materials and Methods

### 2.1. Cell Lines

BJ-5ta cells (ATCC CRL-4001), MRC-5 fibroblasts (ATCC CCL-171), primary mouse embryonic fibroblasts (MEF) [38], immortalized MEFs derived from C57BL/6 mice (CIM (“C57BL/6 immortalized MEF”)) or BALB/c (BIM (“BALB/c immortalized MEF”)) generated by crisis immortalization, as described previously [39], were cultured in Dulbecco’s minimal essential medium (DMEM) supplemented with 10% (*v/v*) FCS, 100 µg/mL streptomycin, 100 U/mL penicillin, and 2 mM glutamine (Gibco, Waltham, MA, USA/Life technologies, Carlsbad, CA, USA). For fluorescence assays, human and mouse cells cell lines were cultured as mentioned above, except CIM cells, which were cultured in DMEM supplemented with 5% (*v/v*) FCS, 100 µg/mL streptomycin, 100 U/mL penicillin, and 2 mM glutamine (Gibco/Life technologies).

### 2.2. Generation of a MLN4924-Resistant BJ-5ta Cell Line

To select an MLN4924-resistant cell line, BJ-5ta cells were continuously passaged in increasing concentrations of MLN4924 for at least 24 weeks. Phenotypical resistance was regularly tested by cell viability assays. Bulk cultures were diluted and plated as single cell clones and cultured in the presence of MLN4924. The experiments shown below were conducted with a cell clone that proliferated in the presence of 10µM MLN4924.

### 2.3. Chemicals

MLN4924 and TAS4464 were purchased from Active Biochem (A-1139) and Med-ChemExpress (HY-128586), respectively.

### 2.4. Immunoblot Analysis

Immunoblotting was performed by lysing cells and adjusting protein concentrations according to the Bradford method, as described before [40]. Cell lysates were separated on SDS-PAGE gels and subsequently transferred to nitrocellulose membranes. Immunoblot analysis was performed as described before [40] using the following antibodies: monoclonal antibody (mAb) anti-ß-actin (A2228, Sigma-Aldrich, St. Louis, MO, USA) and mAb anti-p21 (Santa Cruz sc-6246). Proteins were visualized using peroxidase-coupled secondary antibodies and the substrate SignalFire ECL reagent (Cell Signaling Technology, Danvers, MA, USA).

### 2.5. Cell Viability Assay

Cells were seeded in 96-well plates in 100 µL of medium with or without TAS4464 and MLN4924 and incubated for 72 h. Cell viability was quantified using Orangu cell counting solution (Cell Guidance Systems) according to manufacturer’s instructions.

### 2.6. Viruses, Infection, and Fluorescence Activity Assays

Previously described MCMV and HCMV mutants expressing enhanced green fluorescent protein (eGFP) were employed [23]. BJ-5ta, MLN4924-resistant BJ-5ta (BJ-5ta-R^MLN^), CIM, and MRC-5 cells were seeded in 96-well microplates with black rim for fluorescence determinations. Cells were infected with indicated virus doses (0.1 and 1 PFU/cell), applying centrifugal enhancement (900 g, twice for 15 min). Fluorescence was visualized using a BioSys Bioreader 7000-F-z-i after 24, 48, and 72 h and/or quantified using a microplate multireader (Mithras^2^ LB 943; Berthold Technologies, Bad Wildbad, Germany).

## 3. Results

### 3.1. The Newly Described NAE Inhibitor TAS4464 Stabilizes CRL Target Proteins and Does Not Elicit Overt Cytotoxicity in CMV-Permissive Cell Lines

**Figure 1:**
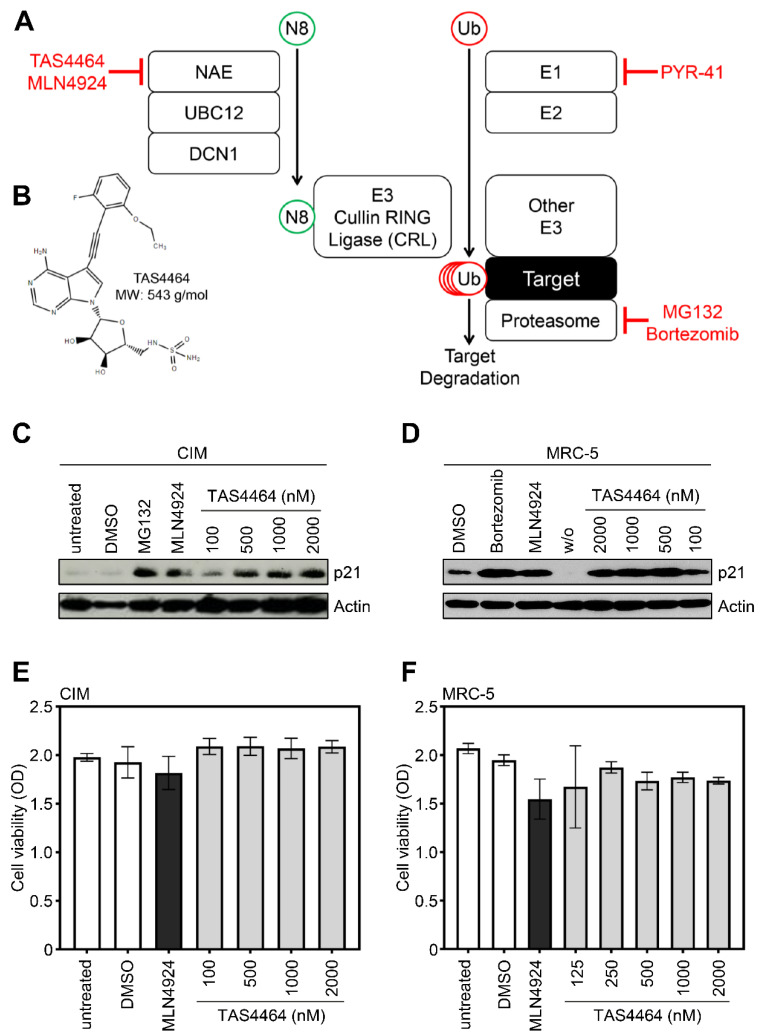
TAS4464 stabilizes CRL targets such as p21, and does not exert overt cytotoxicity. (**A**) Simplified overview of the conjugation of ubiquitin (“Ub”) and Nedd8 (“N8”), as well as target protein degradation through the proteasome. Inhibitors are depicted in red. Please, see the main text of the article for further details and abbreviations. (**B**) The molecular structure of TAS4464 (PubChem CID: 124121823) is shown. (**C**) C57BL/6 immortalized mouse embryonic fibroblasts (“CIM”) and (**D**) human MRC-5 cells were treated with MG132 (1 µM), MLN4924 (5 µM), or the indicated concentrations of TAS4464 (100–2000 nM). Solvent-treated (“DMSO”) and untreated cells served as controls. After 24 h, cells were lysed and subjected to immunoblot analysis to determine the p21 abundance. (**E**) CIM cells were treated with MLN4924 (5000 nM), TAS4464 (100–2000 nM), the solvent (“DMSO”), or were left untreated for 72 h. Afterwards, the cytotoxicity was determined as described in the Materials and Methods section. (**F**) As in (**E**), but human MRC-5 cells were studied.

The NAE inhibitor MLN4924 elicits potent antiviral activity against mouse cytomegalovirus (MCMV) and HCMV [23]. To address if NAE, the known target of MLN4924, is a bona fide HDF of MCMV and HCMV or if MLN4924 might rather act against CMVs through yet unidentified NAE-independent effects, we were eager to test the potential antiviral effects of other NAE inhibitors. Recently, TAS4464 was described as “highly potent and selective inhibitor of NAE” (Ref. [37] and see simplified mechanism and structure in (Figure 1A,B)). The cyclin-dependent kinase inhibitor 1 p21^Cip1/Waf1^ is a well-known target of CRL-mediated ubiquitination and proteasomal degradation [41,42]. Accordingly, the proteasome inhibitor MG-132 and the NAE/CRL inhibitor MLN4924 stabilized p21 in mouse and human cells (Figure 1C,D). Thus, p21 protein abundance levels can serve as a reliable surrogate marker for CRL activity and its inhibition thereof. Consistent with the notion that TAS4464 potently inactivates CRLs, we observed a very pronounced stabilization of p21 upon TAS4464 treatment in mouse and human cells (Figure 1C,D). Since some inhibitors of the UPS or CRLs elicit severe toxicity, preventing meaningful virus replication experiments, we tested the viability of TAS4464-treated cells. These assays showed that TAS4464 does not exert overt cytotoxicity in cell culture (Figure 1E,F), indicating that TAS4464 can be applied to probe into the NAE dependency of MCMV and HCMV replication.

### 3.2. The Novel NAE Inhibitor TAS4464 Elicits Antiviral Activity against Mouse and Human Cytomegalovirus

**Figure 2:**
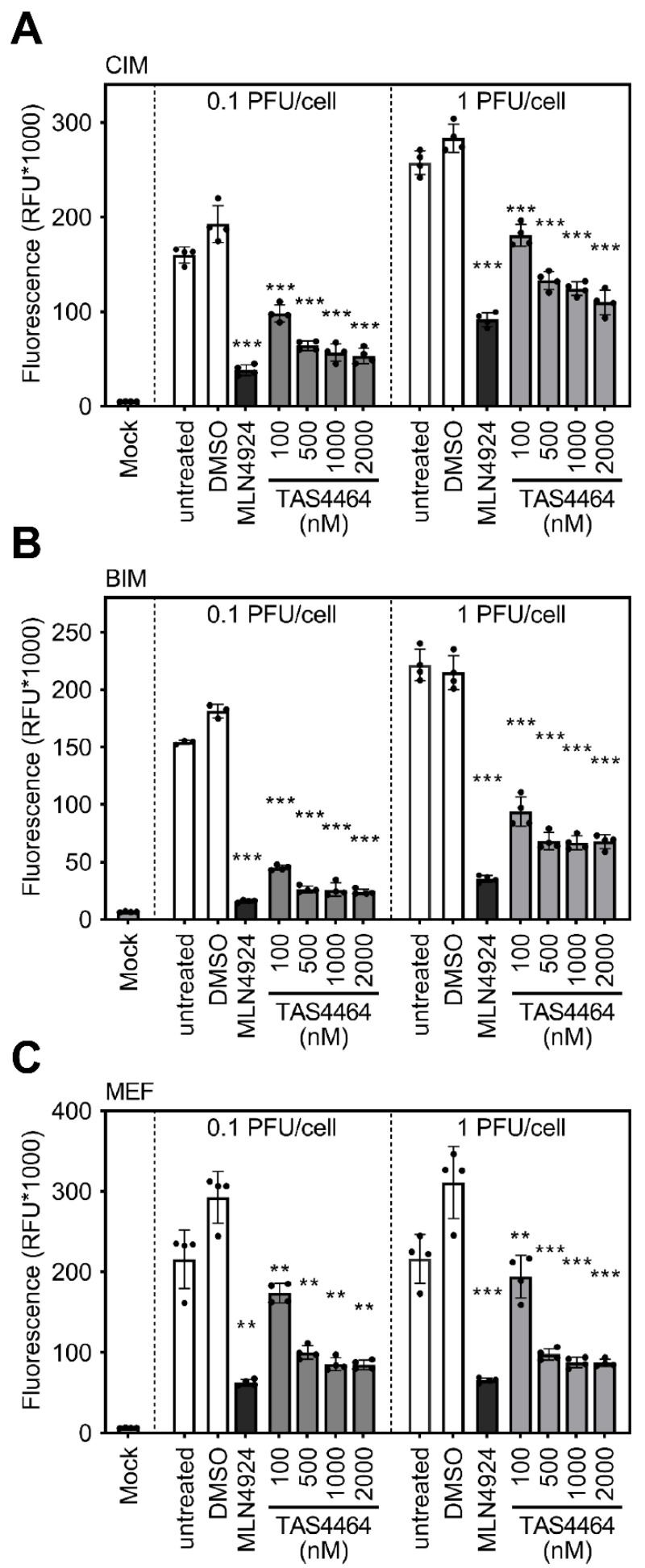
TAS4464 elicits antiviral effect against MCMV in different mouse cells. (**A**) CIM, (**B**) BIM, and (**C**) primary MEF cells were treated with indicated concentrations of TAS4464 (100–2000 nM; shown in grey) or MLN4924 (5000 nM; shown in black) and briefly afterwards infected with MCMV:*eGFP*. Solvent-treated (“DMSO”) and untreated cells served as controls (shown in white). After 3 days, fluorescence was quantified using a microplate multireader. The arithmetic mean ± SD is depicted. Asterisks indicate the level of statistical significance assessed by a two-sided, heteroscedastic *t* test (** *p* < 0.01; *** *p* < 0.001).

To test if TAS4464 elicits antiviral activity against cytomegaloviruses, a previously described immortalized mouse fibroblast cell line (“CIM“; see Materials and Methods section for details) was treated with MLN4924 (5 µM) as positive control or graded TAS4464 concentrations (100–2000 nM) and infected with 0.1 (left panel) or 1 PFU/cell (right panel) of an MCMV reporter mutant expressing the enhanced green fluorescent protein (“MCMV:*eGFP*”). Virus-induced fluorescence was quantified after three days of infection. In agreement with our previous publications, MLN4924 elicited significant antiviral activity (Figure 2A). Please note that accurate dose–response curves for the antiviral activity of MLN4924 against MCMV have been generated previously, indicating antiviral activities at nanomolar concentrations [23]. Consistent with a pronounced antiviral activity, TAS4464 treatment also significantly diminished the MCMV-induced fluorescence compared with untreated or solvent-treated control samples (Figure 2A). The effect of TAS4464 was observed irrespective of the initial dose of infection (0.1 or 1 PFU/cell). Accordingly, TAS4464 and MLN4924 also diminished MCMV-driven reporter gene expression in another immortalized mouse fibroblast cell line (“BIM”) derived from a different mouse strain (BALB/c) (Figure 2B). This antiviral activity was also observed in primary mouse embryonic fibroblasts (Figure 2C), indicating that the antiviral effects elicited by MLN4924 and TAS4464 against MCMV are neither restricted to one mouse strain nor to immortalized cell lines.

**Figure 3:**
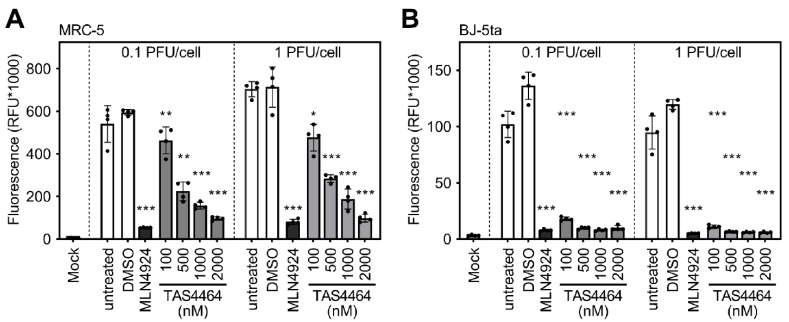
TAS4464 elicits antiviral activity against HCMV in human cells. (**A**) MRC-5 cells were treated with indicated concentrations of TAS4464 (100–2000 nM; shown in grey) or MLN4924 (5000 nM; shown in black) and briefly afterwards infected with HCMV:*eGFP*. Solvent-treated (“DMSO”) and untreated cells served as controls (shown in white). Three days after infection, fluorescence was quantified using a microplate multireader. (**B**) As in (**A**), but the experiment was performed in BJ-5ta cells. The arithmetic mean ± SD is depicted. Asterisks indicate the level of statistical significance assessed by a two-sided, heteroscedastic *t* test (* *p* < 0.05; ** *p* < 0.01; *** *p* < 0.001).

Next, the antiviral effects of MLN4924 and TAS4464 against HCMV were analyzed by a similar approach, applying an eGFP-expressing HCMV reporter virus (“HCMV:*eGFP*”). The HCMV-induced fluorescence was also quantified at 3 days post-infection. In agreement with our previous work, MLN4924 elicited strong antiviral activity and diminished the HCMV-induced fluorophore expression (Figure 3A, black bars). Please note that accurate dose–response curves for the antiviral activity of MLN4924 against HCMV were established in our earlier work, which indicated significant antiviral activity at concentrations as low as 100 nM [23]. Similarly, TAS4464 significantly reduced the HCMV-driven fluorescence compared with untreated and solvent-treated MRC-5 cells (Figure 3A, grey bars). The potent antiviral effect of MLN4924 and TAS4464 was corroborated by similar experiments in the HCMV-permissive fibroblast cell line BJ-5ta (Figure 3B), indicating that two different NAE inhibitory drugs strongly suppress HCMV replication.

### 3.3. MLN4924 and TAS4464 Lose Their Antiviral Activity against HCMV in MLN4924-Resistant Cells

**Figure 4:**
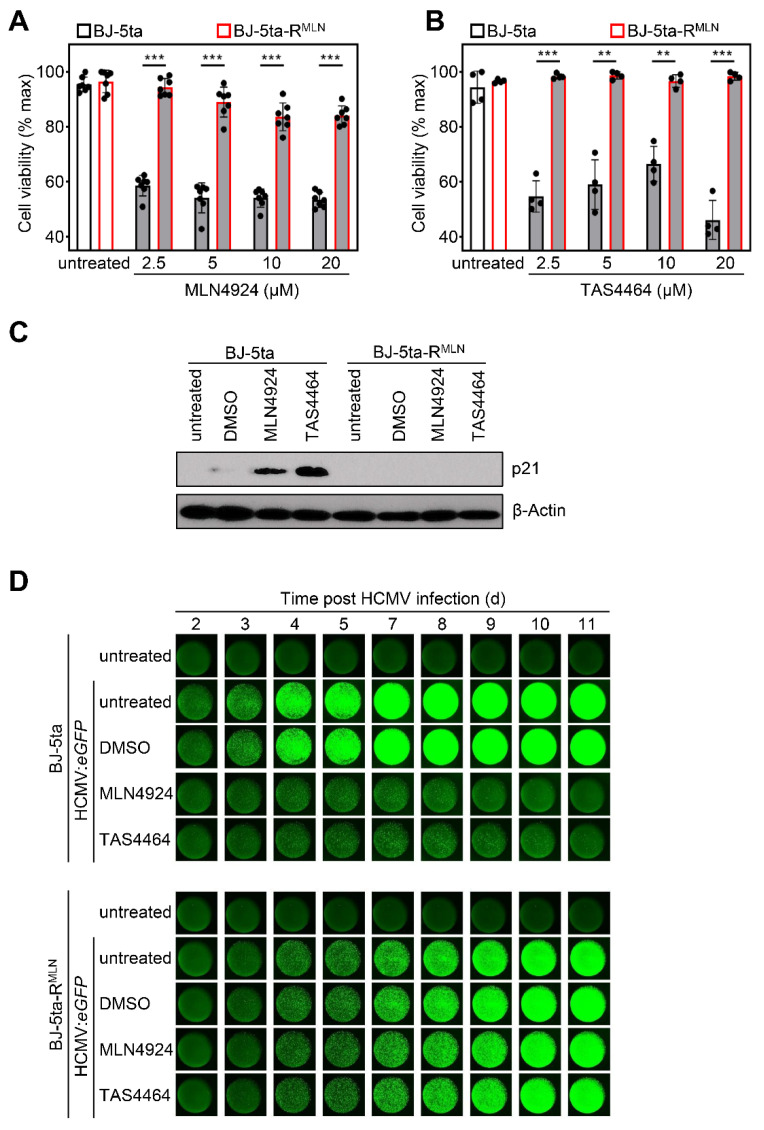
Antiviral effects elicited by MLN4924 and TAS4464 are absent in MLN4924-resistant cells. (**A**) BJ-5ta parental cells and MLN4924-resistant cells (“BJ-5ta-R^MLN^”) were exposed to increasing concentrations of MLN4924 or (**B**) TAS4464 to assess the cell proliferation during a 72 h period. Asterisks indicate the level of statistical significance assessed by a two-sided, heteroscedastic *t* test (** *p* < 0.01; *** *p* < 0.001). (**C**) Parental and BJ-5ta-R^MLN^ cells were left untreated or were treated with DMSO, MLN4924 (5 µM), or TAS4464 (2 µM). Cells were lysed, and the p21 stabilization was assessed by immunoblotting analysis. (**D**) In addition to ruxolitinib (4 µM), BJ-5ta parental and BJ-5ta-R^MLN^ cells were left untreated or were treated with the solvent (DMSO), MLN4924 (5000 nM), or TAS4464 (2000 nM) and briefly afterwards infected with HCMV:*eGFP*. HCMV-induced fluorescence activity was visualized using a Bioreader at indicated time periods after infection. The experiment was conducted in quadruplicate, and one representative well is shown.

As shown above, two different NAE inhibitors elicited antiviral activity against cytomegaloviruses. However, TAS4464—like MLN4924—is to a certain extent related to AMP (see Figure 1B). Thus, we wondered if direct virus-encoded targets of MLN4924 and TAS4464 beyond NAE may exist. To address this, an MLN4924-resistant cell clone based on the HCMV-permissive BJ-5ta cell line was selected by continuous cell culture passage in the presence of increasing concentrations of MLN4924. While the proliferation of parental BJ-5ta cells is significantly diminished in the presence of graded MLN4924 concentrations, the resistant cell clone (“BJ-5ta-R^MLN^”) had acquired the capacity to proliferate even at high (20 µM) MLN4924 concentrations (Figure 4A). Intriguingly, BJ-5ta-R^MLN^ cells also acquired resistance to TAS4464 in terms of proliferation (Figure 4B). This finding suggests that the inhibitory function related to the binding of both drugs to NAE constitutes the dominant determinant of anti-proliferative effects of these drugs. Furthermore, NAE mutations selected during continuous MLN4924 exposure seem to confer cross-resistance against TAS4464. In accord with the resistance of BJ-5ta-R^MLN^ in terms of anti-proliferative effects, MLN4924 and TAS4464 failed to stabilize the CRL target protein p21, while the stabilization was observed in the parental cells (Figure 4C).

To evaluate if MLN4924 and TAS44964 elicit their antiviral activity through the inhibition of NAE-, neddylation-, and CRL-dependent effects, or through virus-encoded targets, or through a combination of both, we compared the antiviral effects against HCMV in BJ-5ta parental and BJ-5ta-R^MLN^ cells. As expected, BJ-5ta cells were highly permissive for HCMV, and MLN4924 as well as TAS4464 elicited potent antiviral effects against HCMV:*eGFP* in terms of reducing the virus-induced fluorescence in BJ-5ta cells (Figure 4D and data not shown). Conversely, BJ-5ta-R^MLN^ cells showed a diminished HCMV permissiveness irrespective of the application of NAE inhibitory drugs (data not shown). Although this may be regarded as an argument in favor of the hypothesis that NAE is an HDF for HCMV and that NAE alterations selected during resistance acquisition impair viral replication, we cannot formally exclude second-site mutations or other clonal effects. To enhance the HCMV permissiveness of BJ-5ta-R^MLN^ cells, we used a pharmacologic approach. We added the Janus kinase (JAK) inhibitor ruxolitinib, which we previously found to foster cytomegalovirus replication by blunting interferon signaling [43]. In the presence of ruxolitinib, the HCMV-induced fluorescence expression in BJ-5ta-R^MLN^ cells was enhanced (data not shown). Intriguingly, all antiviral effects elicited by MLN4924 and TAS4464 in parental BJ-5ta cells were lost in BJ-5ta-R^MLN^ cells (Figure 4D, lower panel), indicating that MLN4924 and TAS4464 act as antivirals against HCMV by targeting the host-derived factor NAE. Taken together, these data reveal that the direct effects of the drugs MLN4924 and TAS4464 on viral proteins either do not exist at all or at least do not play a relevant role during virus replication in fibroblasts.

## 4. Discussion

We demonstrate here that nanomolar concentrations of the NAE inhibitor TAS4464 stabilizes CRL substrates such as p21 and elicits significant and dose-dependent antiviral activity in different cells against mouse and human CMV. These findings support and extend our previous findings that showed that the drug MLN4924, which—like TAS4464—inhibits NAE-dependent neddylation and neddylation-dependent CRL activity, also elicits broad and potent antiviral activity. The IC_50_ of TAS4464 against NAE is approximately 11-fold superior to MLN4924 [37]. In addition, the inhibitory off-target effect of TAS4464 on carbonic anhydrase II (CA2) is approximately 43-times reduced. Thus, TAS4464 seems to be more potent and more specific than MLN4924.

Since two different CRL activity inhibitors elicit potent antiviral activity against HCMV, this may suggest that any efficient pharmacologic blockade of NAE or CRL activity could diminish cytomegalovirus replication. Given that neddylation and CRL inhibitors emerge as an area of very active research in the field of tumor drug development, it is tempting to speculate that upcoming compounds that target neddylation or CRL activity could be repurposed and applied as antiviral drugs against cytomegaloviruses.

Previously, others have raised MLN4924-resistant cell clones during continuous passage in increasing MLN4924 concentrations. In these cases, mutations in the adenosine triphosphate binding pocket and Nedd8-binding cleft of the beta subunit of NAE conferred the treatment-emergent resistance to MLN4924 in three independent cell lines [44]. Especially, mutations that result in an exchange of the alanine at position A171 to bulkier amino acids such as threonine or aspartic acid conferred resistance to MLN4924. This fits very well with structural modelling in which A171T/D would result in a clash with the indane “base-like” group of MLN4924 [44]. We applied a comparable approach with a HCMV-permissive fibroblast cell line. Intriguingly, we found that the cell clone selected for MLN4924 resistance turned out to also be resistant against TAS4464. To our knowledge, such co-resistance has not been shown before, but it fits well with the aforementioned structural explanation because the 1-ethoxy-2-ethynyl-3-fluorobenzen group of TAS4464 (Figure 1B) seems to fulfil a comparable function and occupy a similar space in NAEβ as the indane group of MLN4924.

In contrast with parental cells, neither MLN4924 nor TAS4464 elicit antiviral activity in cells that had been forced to acquire MLN4924 resistance and TAS4464 co-resistance. This finding strongly suggests that NAE and NAE-dependent events such as the ubiquitination catalyzed by CRLs and subsequent proteasomal degradation of CRL substrates constitute bona fide HDFs that play indispensable roles for MCMV and HCMV replication. In the future, it will be very interesting to investigate which viral and/or cellular proteins need to be ubiquitinated in order to allow efficient cytomegalovirus replication. In HCMV-infected cells, such candidate proteins should be among the proteins being regulated in a post-translational manner [45], proteins increased upon inhibitors of the proteasome [31,46], proteins stabilized by inhibitors of CRLs [23], or among viral CRL- or *DDB1 and cullin-associated factor* (DCAF)-binding proteins [31,32,34,35,36,47].

Recent data indicate that some non-cullin proteins are also modified by neddylation (reviewed in [48]). Even some viral proteins have been shown to be subjected to Nedd8 conjugation (see e.g., [49,50]). Thus, it is also conceivable that the drug-induced lack of neddylation of viral and/or host-derived proteins other than cullins contribute to the antiviral activity elicited by MLN4924 and TAS4464. Although it violates Occam’s parsimony razor, we cannot exclude that CRLs regulate the half-life of proteins, which in turn modify another layer of downstream mediators (e.g., by posttranslational modification), and that the latter affect cytomegalovirus replication.

Based on our cytotoxicity assessment in conjunction with the ongoing clinical trials administering MLN4924 to patients, we concluded that the antiviral effect is most likely not a consequence of cytotoxicity. However, it should be emphasized that both drugs—as is to be expected from anti-cancer drugs—elicit profound anti-proliferative effects. Given that some viruses (e.g., simple retroviruses) exclusively replicate in proliferating cells and that other viruses “prefer” cells being situated at defined phases of the cell cycle (e.g., the pre-S phase), it is conceivable that the anti-proliferative effects of MLN4924 and TAS4464 contribute to the antiviral effects. We actively tried to minimize the contribution of the anti-proliferative activity on the antiviral effects by conducting the experiments using confluent cell layers in which most cells have arrested their cell cycle due to contact inhibition. If non-confluent cell layers were exposed to NAE or UPS inhibitors and infected, the observed antiviral effects were usually far more drastic (data not shown); however, this may simply be a consequence of the obvious fact that fewer cells produce fewer virus progeny. The precise contribution of anti-proliferative effects to the antiviral effect of CRL/UPS inhibitors is difficult to quantify. We assume that this will only be possible once specific inhibitors have been developed that spare CRLs that are essential for cell cycle regulation. From our perspective, however, the multitude of HCMV-encoded proteins directly or indirectly interacting with CRLs (e.g., pUL145, pM27, pRL1, and other currently under investigation) rather supports the notion that cytomegaloviruses actively exploit CRLs and that their pharmacologic blockade deprives the virus of necessary aspects of host cell modulation and exploitation.

## Data Availability

All data are part of the manuscript.
